# Diagnostic value of 24-h urinary aldosterone and related biomarkers for screening primary aldosteronism in medication-exposed populations

**DOI:** 10.3389/fendo.2026.1792072

**Published:** 2026-02-27

**Authors:** Siyu Chen, Ke Chen, Junyi Wu, Jiashu Yang, Linlin Shen, Hui Yuan

**Affiliations:** Department of Clinical Laboratory, Beijing Anzhen Hospital, Capital Medical University, Beijing, China

**Keywords:** aldosterone-to-renin ratio, hypertension, medication effect, primary aldosteronism, urinary aldosterone

## Abstract

**Objective:**

To evaluate the screening value of 24-hour urinary aldosterone (24h-Uald) and the urinary aldosterone–to–renin ratio (UARR) for primary aldosteronism (PA in hypertensive patients under conditions of ongoing antihypertensive medication use.

**Methods:**

Hypertensive patients who underwent PA screening at our hospital between August 2024 and August 2025 were retrospectively enrolled. Baseline clinical characteristics and biochemical parameters were collected while patients were receiving ongoing antihypertensive therapy. Multivariable logistic regression analysis was performed to assess the independent associations of 24h-Uald and UARR with PA. Diagnostic performance was evaluated using receiver operating characteristic (ROC) curve analysis.

**Results:**

Both 24h-Uald and UARR levels were significantly higher in patients with PA than in those with essential hypertension (EH) (P < 0.05). After adjustment for potential confounders, including age, body mass index, renal function, and history of coronary artery disease, 24h-Uald and UARR remained independently associated with PA. ROC curve analysis demonstrated a modest diagnostic performance for 24h-Uald (AUC = 0.657), whereas UARR exhibited excellent diagnostic accuracy (AUC = 0.862).

**Conclusions:**

Under conditions of ongoing antihypertensive medication use, the diagnostic performance of 24h-Uald alone for PA screening is limited. In contrast, UARR, incorporating renin measurements, demonstrates higher diagnostic accuracy and greater clinical utility.

## 1 Introduction

For Primary aldosteronism (PA) is one of the most common causes of secondary hypertension and is characterized by inappropriate aldosterone overproduction, leading to a spectrum of clinical manifestations, including hypertension and hypokalemia (1). Excessive and inappropriate aldosterone secretion promotes cardiovascular remodeling and increases the risk of adverse cardiovascular events, such as atrial fibrillation and heart failure, which occur more frequently in patients with primary aldosteronism than in those with essential hypertension (2–4). In recent years, with the increasing understanding of PA, the clinical screening rate has been increasing year by year and is currently around 5% (5). Early screening and diagnosis of primary aldosteronism enable timely individualized treatment and may reduce the risk of disease-related complications.

The renin–angiotensin–aldosterone system (RAAS) plays a central role in maintaining fluid balance, regulating electrolyte homeostasis, and controlling blood pressure. The pathophysiology of PA is characterized by autonomous aldosterone secretion from the adrenal cortex, which leads to feedback suppression of renin release. Consequently, initial screening for PA relies primarily on the aldosterone-to-renin ratio (ARR), which reflects the inappropriate elevation of aldosterone relative to suppressed renin levels (6). This index provides a comprehensive response to the status of the RAAS axis. However, plasma aldosterone levels are susceptible to a wide range of medications and physiological factors (7), and therefore medication adjustments, postural and salt intake control, and other treatments are often required prior to screening. Multiple antihypertensive medications may result in false-positive or false-negative ARR, which in turn affects the judgment of PA (8). Current guidelines recommend that all hypertensive patients be screened for PA at least once. Interfering medications need to be discontinued for 2–4 weeks, but discontinuation may lead to uncontrolled blood pressure and delayed diagnosis, so the necessity of switching medications before screening for ARR remains a controversial issue in clinical practice. Aldosterone secretion has a distinct circadian rhythm. Compared with a single blood sample, 24-h urinary aldosterone (24h-Uald) provides a more complete picture of a complete secretion cycle, while the urine matrix is relatively clean and less disturbed by external factors (9). Previous studies suggest that 24h-Uald has potential clinical value in the diagnosis of PA (10–12).

Based on the above background, the aim of this study was to investigate the value of 24h-Uald and its related indexes in PA screening under the condition of taking drugs that interfere with the reliability of ARR results.

## 2 Materials and methods

### 2.1 Study design and patient population

This retrospective study was conducted at Beijing Anzhen Hospital, Capital Medical University. A total of 280 hypertensive patients who underwent screening for primary aldosteronism (PA) between August 2024 and August 2025 were included.

Inclusion criteria were as follows (1): Patients who met the diagnostic criteria for hypertension, defined as systolic blood pressure >140 mmHg and/or diastolic blood pressure >90 mmHg on three separate occasions on non-consecutive days in the absence of antihypertensive medications (2). Patients receiving medications that could interfere with the aldosterone-to-renin ratio (ARR) at admission and who had not yet undergone medication washout (3). Patients who subsequently completed medication washout and underwent repeat ARR screening and confirmatory testing for PA (4). Patients with complete clinical and laboratory data.

Exclusion criteria were as follows: (1) Missing key clinical or laboratory data. (2) Presence of other causes of secondary hypertension, including renal parenchymal hypertension, renal vascular hypertension, pheochromocytoma, and Cushing’s syndrome. (3) Failure to complete medication washout and subsequent PA screening and confirmatory testing. (4) Pregnancy. (5) Severe comorbid conditions, including advanced hepatic or renal failure, severe cardiovascular or cerebrovascular disease, autoimmune disease, or malignancy.

Interfering medications and washout: We recorded detailed medication profiles for all patients at admission (pre-washout). Medications were categorized into three groups based on their potential pharmacological interference with the ARR:

Potential false-negative group, including angiotensin-converting enzyme inhibitors (ACEIs), angiotensin receptor blockers (ARBs), calcium channel blockers (CCBs), diuretics, mineralocorticoid receptor antagonists (MRAs, e.g., spironolactone), and amiloride (13). Potential false-positive group, including β-blockers and non-steroidal anti-inflammatory drugs (NSAIDs) (14). Mixed-interference group, referring to patients concurrently taking medications from both aforementioned categories.

To ensure diagnostic accuracy, specific washout periods were implemented: MRAs and diuretics were discontinued for at least 4 weeks, while other interfering medications (including ACEIs, ARBs, CCBs, and β-blockers) were discontinued for at least 2 weeks prior to biochemical testing and confirmatory trials (15).

Confirmatory testing: Patients underwent either the captopril challenge test (CCT) or the saline infusion test (SIT). For the CCT, 50 mg of captopril was administered orally, and blood samples were collected after 4 hours. PA was diagnosed based on a comprehensive assessment: aplasma aldosterone concentration (PAC) decrease of ≤30% from baseline and/or a post-CCT PAC≥ 11ng/dL. For the SIT, 2000 mL of 0.9% saline was infused intravenously over 4 hours, and a post-infusion PAC >10 ng/dL supported the diagnosis. Patients were classified into the PA or essential hypertension (EH) group based on the results of confirmatory testing.

### 2.2 Clinical and biochemical data

Clinical and biochemical data were retrieved from the hospital electronic medical record system. Baseline clinical information at admission (prior to medication washout) was collected, including sex, age, body mass index (BMI), systolic and diastolic blood pressure, and comorbidities such as hyperlipidemia, coronary heart disease (CHD), and diabetes mellitus. Biochemical parameters at admission were also recorded, including urea, creatinine, estimated glomerular filtration rate (eGFR), uric acid, serum potassium (K), plasma aldosterone concentration (PAC), direct renin concentration (DRC), aldosterone-to-renin ratio (ARR), 24h-Uald, 24-h urinary potassium, and 24-h urinary sodium. Boric acid was added to the 24-hour urine collection containers as a preservative to maintain the stability of aldosterone levels prior to analysis.

The urinary aldosterone-to-renin ratio (UARR) was calculated using the following formula: UARR = 24h-Uald/DRC.

Here, 24-h Uald represents the 24-hour urinary aldosterone level (numerator), and DRC denotes the plasma direct renin concentration (denominator).

### 2.3 Statistical analysis

Sample size calculation was performed using PASS software, and the adequacy of the sample size was determined based on the calculated statistical power. Data analysis was conducted using R software (version 4.4.1). The normality of continuous variables was assessed using the Shapiro–Wilk test. Normally distributed continuous variables were expressed as mean ± standard deviation (mean ± SD), whereas non-normally distributed continuous variables were presented as median (P25, P75). Categorical variables were expressed as counts and percentages. Differences between two groups for continuous variables were compared using the independent samples t-test or the Mann–Whitney U test, as appropriate, and categorical variables were compared using the chi-square test. The associations between diagnostic indicators and clinical parameters were assessed using Spearman’s rank correlation analysis. Multivariable logistic regression analysis was used to assess the association between 24-h urinary aldosterone and related indicators with PA. The diagnostic performance of each indicator was evaluated using receiver operating characteristic (ROC) curve analysis, and differences in area under the curve (AUC) between ROC curves were compared using the DeLong test.

## 3 Results

### 3.1 Baseline characteristics of patients with PA and EH

**Figure 1:**
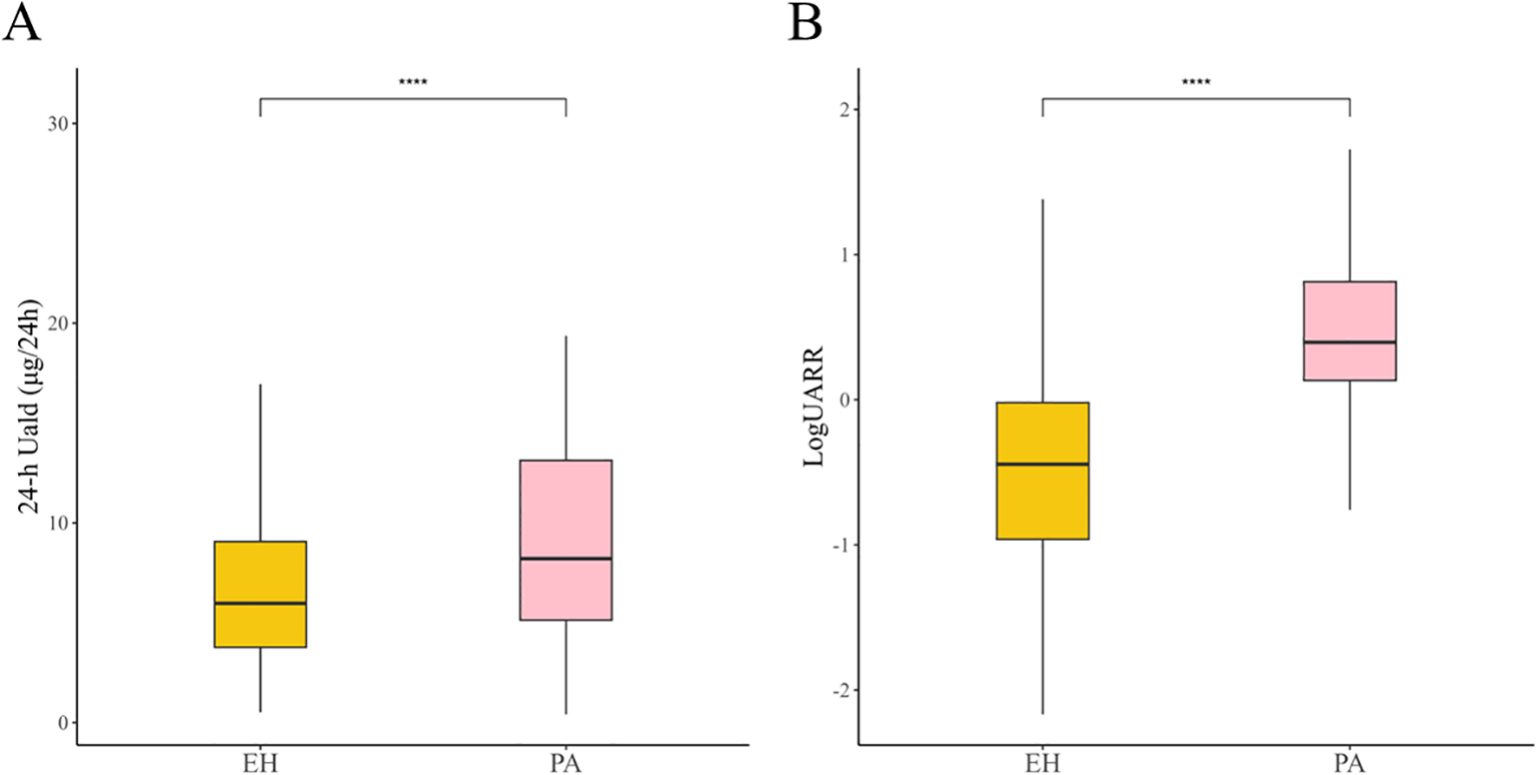
Differences in 24-h Uald levels and UARR between PA and EH patients. **(A)** Comparison of 24-h Uald levels between PA and EH patients. **(B)** Comparison of log-transformed UARR between PA and EH patients. Ratios were log-transformed for graphical clarity.

A total of 280 hypertensive patients who had not yet undergone medication washout and were awaiting diagnosis were included in this study. After medication washout, 204 patients were diagnosed with EH and 76 patients were diagnosed with PA. The baseline characteristics of the patients are presented in **Table 1**. Regarding medication status at admission, no significant differences were observed between the PA and EH groups in the distribution of potential false-negative, potential false-positive, and mixed-interference medications (P = 0.219). Compared with the EH group, patients with PA were older and had higher rates of diabetes and CHD. In addition, PAC, ARR, K, 24h-Uald, and the UARR were significantly higher in the PA group, while BMI, eGFR, and renin levels were significantly lower (P < 0.05). There were no significant differences between the two groups in terms of sex, systolic blood pressure, diastolic blood pressure, history of hyperlipidemia, urea, creatinine, uric acid, 24-h urinary sodium, or 24-h urinary potassium. The differences in 24-h Uald and log-transformed UARR between PA and EH patients are shown (**Figure 1**).

**Table 1:** Clinical and biochemical characteristics of patients with PA and EH.

Characteristics	EH (n=204)	PA(n=76)	*P value*
Age (y)	52.00 (42.00, 61.00)	55.00 (47.75, 62.00)	0.03454
Male	126(61.8%)	39(51.3%)	0.114
BMI (kg/m^2^)	27.70 (24.87, 30.50)	26.00 (24.00, 28.75)	0.04549
SBP (mm Hg)	148.00 (134.00, 162.25)	150.50 (139.00, 166.50)	0.3504
DBP (mm Hg)	96.06 ± 15.62	95.97 ± 13.14	0.9615
Hyperlipidemia	113(55.4%)	50(65.8%)	0.1167
Diabetes	30(14.7%)	19(25%)	0.0438
CHD	8(3.9%)	12(15.8%)	<0.01
Urea(mmol/L)	5.38 (4.53, 6.26)	5.38 (4.75, 6.51)	0.2672
Creatinine (μmol/L)	71.05 (59.25, 81.58)	71.65 (59.45, 82.22)	0.4978
eGFR (mL/min/1.73 m^2^)	97.34 (89.27, 108.27)	95.18 (81.61, 101.72)	0.01328
Uric acid (μmol/L)	363.05 (295.75, 430.75)	335.50 (276.00, 414.48)	0.07645
K^+^(mmol/L)	3.93 ± 0.33	3.79 ± 0.42	<0.01
PAC (ng/dl)	12.50 (8.25, 17.92)	17.70 (13.02, 24.97)	<0.01
DRC (ulU/ml)	17.75 (6.10, 38.67)	3.50 (1.15, 6.65)	<0.01
ARR	0.80 (0.20, 1.80)	5.25 (2.58, 11.45)	<0.01
24h- Uald (μg/24h)	5.95 (3.75, 9.02)	8.21 (5.13, 13.13)	<0.01
24h- Urine Na^+^ (mmol/24h)	123.55 (83.90, 171.77)	114.50 (86.18, 152.65)	0.3813
24-h Urine K^+^ (mmol/24h)	37.60 (30.90, 49.10)	37.60 (31.32, 46.58)	0.7312
UARR	0.36(0.11, 0.93)	2.49(1.36, 6.52)	<0.01
Medication Status			0.219
Potential false-negative drug ^a^	120(58.82%)	36(47.37%)	
Potential false-positive drug^b^	17(8.33%)	9(11.84%)	
Mixed-interference drug^c^	67(32.84%)	31(40.79%)	

a Potential false-negative drugs include ACEIs, ARBs, CCBs, diuretics, MRAs, and amiloride.

b Potential false-positive drugs include β-blockers and NSAIDs.

c Mixed-interference group refers to the concurrent use of medications from both of the aforementioned categories.

### 3.2 Association between 24-h Uald, UARR and the risk of PA

To evaluate the biochemical stability of the indicators, we first analyzed the correlations between 24h-Uald, UARR, and renal-related clinical parameters. Spearman correlation analysis showed that 24h-Uald was significantly correlated with serum K (rho = -0.25, P < 0.01) and marginally correlated with eGFR (rho = 0.12, P = 0.052). In contrast, UARR demonstrated superior stability, showing no significant association with serum K (P = 0.18) and only a very weak correlation with eGFR (rho = -0.12, P = 0.045). These findings suggest that UARR is more robust against fluctuations in serum potassium and renal function than 24h-Uald.

Multivariable logistic regression analysis was performed to identify factors associated with PA (**Table 2**). Model 1 included age, BMI, history of CHD, eGFR, and K. The results showed that BMI(OR 0.93, 95% CI 0.86–1.00, P = 0.04), CHD history (OR4.10, 95% CI1.49–11.32, P < 0.01), eGFR (OR 0.97, 95% CI0.95–0.99, P =0.01) and K(OR 0.23, 95% CI 0.10–0.51, P< 0.01) were significantly associated with PA, whereas age was not statistically significant. In Model 2, after 24h-Uald was added to Model 1, 24h-Uald remained an independent predictor of PA (OR1.17, 95% CI1.09–1.26, P < 0.01), while BMI, CHD history, eGFR and K continued to be independent factors. In Model 3, UARR was added to Model 1, UARR was independently and positively associated with PA (OR1.38, 95% CI1.19–1.59, P < 0.01), while CHD history, eGFR and K remained statistically significant in this model.

**Table 2:** Clinical and biochemical characteristics in relation to PA.

Variable	Model 1		Model 2		Model 3	
	OR	*P value*	OR	*P value*	OR	*P value*
Age	1.00(0.95-1.03)	0.76	1.02(0.98-1.05)	0.40	0.99 (0.96–1.03)	0.59
BMI	0.93(0.86-1.00)	0.04	0.88(0.81-0.96)	<0.01	0.95(0.88-1.03)	0.24
CHD	4.10(1.49-11.32)	<0.01	2.94(1.00-8.64)	0.05	4.20(1.42-12.37)	0.01
eGFR	0.97(0.95-0.99)	0.01	0.97(0.95-1.00)	0.02	0.97(0.95-1.00)	0.02
K	0.23(0.10-0.51)	<0.01	0.37(0.16-0.88)	0.02	0.29(0.12-0.71)	<0.01
24-h Uald			1.17(1.09-1.26)	<0.01		
UARR					1.38(1.19-1.59)	<0.01

### 3.3 Diagnostic value of 24-h Uald and UARR for PA

**Figure 2:**
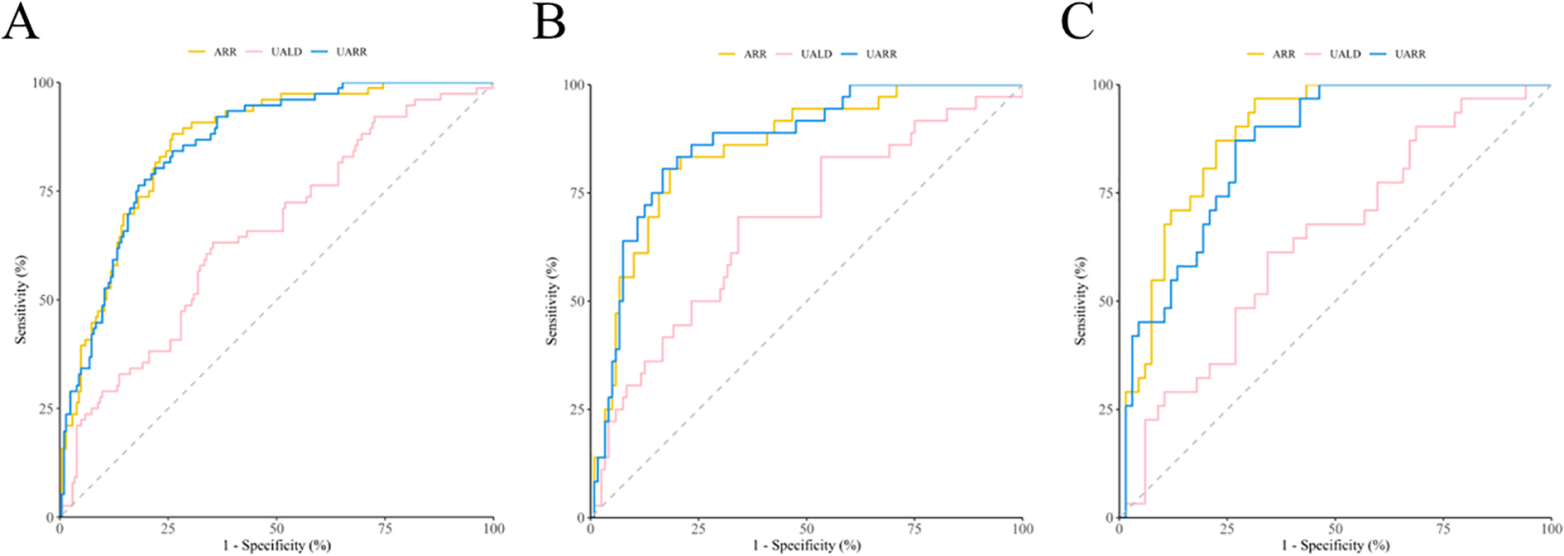
ROC curves of 24-h Uald, UARR, and ARR for the diagnosis of PA. **(A)** ROC curves without stratification by antihypertensive medications. **(B)** ROC curves in patients receiving potential false-negative medications. **(C)** ROC curves in patients receiving mixed medications (containing both false-negative and false-positive effects).

ROC curves for ARR, 24h-Uald, and the UARR were plotted without stratifying patients by medication status (**Figure 2A**). The areas under the ROC curves (AUCs) for 24h-Uald, UARR, and ARR were 0.657, 0.862, and 0.867, respectively, indicating that UARR had good discriminative ability. The AUC of UARR was comparable to that of ARR, with no statistically significant difference (P = 0.618). The corresponding optimal cut-off values, sensitivities, and specificities are presented in **Table 3**. Using the Youden index to determine the optimal thresholds, the cut-off value for 24h-Uald was 7.435 μg/24 h (sensitivity 0.632, specificity 0.652), the cut-off value for UARR was 0.893 (sensitivity 0.833, specificity 0.791), and the cut-off value for ARR was 1.65 (sensitivity 0.882, specificity 0.740).

**Table 3:** Diagnostic performance of 24-h Uald, UARR, and ARR for diagnosing PA.

Medications	Biomarker	AUC	Threshold	Sensitivity	Specificity
Overall medication use	ARR	0.867	1.650	0.882	0.740
	24-h Uald (μg/24h)	0.657	7.435	0.632	0.652
	UARR	0.862	0.893	0.842	0.745
False-negative medications	ARR	0.863	1.450	0.833	0.791
	24-h Uald (μg/24h)	0.688	7.900	0.694	0.667
	UARR	0.874	0.893	0.806	0.842
Confounding medications	ARR	0.90	2.45	0.90	0.76
	24-h Uald (μg/24h)	0.654	7.42	0.61	0.67
	UARR	0.871	1.32	0.87	0.75

### 3.4 Baseline characteristics of patients with PA and EH under the influence of different types of drugs

All patients included in this study were evaluated before medication washout. Since most patients were taking multiple antihypertensive drugs, medications were categorized into three groups based on their potential interference with ARR results: false-negative drugs, false-positive drugs, and mixed-interference drugs (i.e., drugs with both false-negative and false-positive effects). Because the sample size in the false-positive drug group was limited, this group was not analyzed.

Clinical characteristics of PA and EH patients under false-negative and mixed-interference drug conditions are shown in **Table 4**. Under false-negative drug conditions, PAC, ARR, 24h-Uald, 24-h urinary potassium, and UARR were significantly higher in the PA group than in the EH group, while the prevalence of hyperlipidemia, renin levels, and 24-h urinary sodium were significantly lower in PA (P < 0.05). Under mixed-interference drug conditions, patients with PA were older and had higher rates of diabetes and coronary heart disease. In addition, PAC, ARR, 24h-Uald, and UARR were significantly higher in the PA group, whereas eGFR, uric acid, and renin levels were significantly lower compared with the EH group (P < 0.05).

**Table 4:** Clinical and biochemical characteristics of Patients with PA and EH stratified by drug.

	False-negative medications (n= 156)			Confounding medications (n= 98)		
	EH(n=121)	PA(n=35)	*P value*	EH(n=67)	PA(n=31)	*P Value*
Age	51(41, 61.25)	53(45, 60.25)	0.60	52.72 ± 11.74	57.55 ± 9.69	0.05
Mela (man)	75(62.5%)	18(50%)	0.25	41(61.2%)	19(61.3%)	1.00
BMI	27.65(24.75, 30.70)	26.25(23.70, 28.48)	0.18	27.93 ± 4.06	26.64 ± 3.70	0.14
SBP	149.50(139.75, 162.25)	148.50(138.00, 168.25)	0.82	144.00 ± 22.88	152.19 ± 20.27	0.10
DBP	97(87.75, 105.25)	98(85, 108)	0.74	93.81 ± 17.52	94.32 ± 13.03	0.87
Hyperlipidemia	55(45.8%)	23(63.9%)	0.09	46(68.7%)	21(67.7%)	1.00
Diabetes	15(12.5%)	7(19.4%)	0.44	10(14.9%)	10(32.3%)	0.08
Coronary heart Disease	5(4.2%)	2(5.6%)	0.66	3(4.5%)	7(22.6%)	0.01
Urea(mmol/L)	5.30(4.45, 6.10)	5.42(4.95, 6.22)	0.29	5.51(4.74, 6.50)	5.39(4.72, 6.83)	0.90
Creatinine (μmol/L)	70.80(58.83, 81.98)	70.30(63.63,84.20)	0.61	71.08 ± 14.39	74.65 ± 16.87	0.28
eGFR (mL/min/1.73 m^2^)	98.87(90.30, 107.83)	97.33(79.500.04, 105.36)	0.15	96.70 ± 13.42	90.26 ± 13.32	0.03
Uric acid (μmol/L)	350.85(292.98, 424.13)	343.00(290.30, 399.45)	0.51	382.29 ± 95.03	341.85 ± 88.59	0.05
K^+^(mmol/L)	3.95(3.74, 4.17)	3.85(3.65, 4.08)	0.13	3.88 ± 0.39	3.75 ± 0.36	0.13
Pac(ng/dl)	13.25(8.68, 18.05)	17.85(13.40, 22.43)	<0.01	10.00(7.75, 17.95)	17.80(13.85, 27.55)	<0.01
DRC (ulU/ml)	22.80(10.08, 52.40)	4.50(2.45, 9.43)	<0.01	14.80(3.60, 32.85)	2.00(0.75, 4.70)	<0.01
ARR	0.70(0.20, 1.30)	3.80(1.78, 5.90)	<0.01	0.80(0.35, 2.30)	7.60(4.70, 19.70)	<0.01
24h- Uald(μg/24h)	6.05(3.86, 9.30)	9.36(5.89, 13.75)	<0.01	5.62(3.42, 8.56)	8.20(4.61, 12.38)	0.01
24h- Urine Na(mmol/24h)	126.85(86.15, 171.10)	117.85(88.95, 182.65)	<0.01	122.20(83.20, 176.05)	113.6(79.30, 129.90)	0.29
24h- Urine K(mmol/24h)	38.30(32.15, 45.73)	42.20(32.23, 55.50)	0.35	37.00(29.00, 50.05)	36.00(31.15, 43.95)	0.65
UARR	0.31(0.09, 0.70)	1.80(1.00, 3.35)	<0.01	0.41(0.13, 1.41)	3.24(1.69, 10.11)	<0.01

### 3.5 Diagnostic value of UARR and 24-h Uald under the influence of different types of drugs

ROC curves for ARR, 24h-Uald, and the UARR were plotted separately for each medication category (**Figures 2B, C**). The corresponding optimal cut-off values, sensitivities, and specificities are shown in **Table 3**. In the false-negative drug interference group, the AUCs for 24h-Uald, UARR, and ARR were 0.688, 0.874, and 0.863, respectively. UARR demonstrated good diagnostic performance, and its AUC was comparable to that of ARR, with no statistically significant difference (P = 0.381). Using the Youden index, the optimal cut-off values were 7.900 μg/24 h for 24h-Uald (sensitivity 0.694, specificity 0.667), 0.893 for UARR (sensitivity 0.806, specificity 0.842), and 1.45 for ARR (sensitivity 0.833, specificity 0.791).

In the mixed-interference drug group, the AUCs for 24h-Uald, UARR, and ARR were 0.654, 0.871, and 0.900, respectively. UARR also demonstrated good diagnostic performance, and its AUC was comparable to that of ARR, with no statistically significant difference (P = 0.113). Using the Youden index, the optimal cut-off values were 7.420 μg/24 h for 24h-Uald (sensitivity 0.610, specificity 0.670), 1.32 for UARR (sensitivity 0.870, specificity 0.750), and 2.45 for ARR (sensitivity 0.900, specificity 0.760).

## 4 Discussion

In this study, we evaluated the predictive value of 24-h Uald and UARR for PA screening in patients who had not undergone medication washout. The results showed that 24-h urinary aldosterone alone had limited diagnostic performance in distinguishing PA from EH, whereas UARR demonstrated better discriminative ability.

Whether to switch antihypertensive medications before PA screening has long been a focus of clinical debate. Some patients, due to comorbid cardiovascular diseases or refractory hypertension, are unable or unsuitable to undergo medication adjustment (16). Notably, pivotal studies have demonstrated that the ARR remains a robust and accurate screening tool even in patients maintaining their antihypertensive medications (17, 18). This established reliability of ratio-based assessments provides a strong theoretical foundation for the clinical application of UARR without medication washout. However, despite the robustness of the ratio, plasma aldosterone remains susceptible to various influencing factors such as momentary body position and sodium intake. Under conditions without medication adjustment, these factors may increase the difficulty of interpreting screening results (7). Furthermore, plasma biomarkers are highly sensitive to the patient’s acute state at the moment of sampling. Markou et al. (19) demonstrated that acute psychological or physiological stress can trigger transient aldosterone hypersecretion via the ACTH pathway, potentially leading to fluctuations in the ARR. In contrast, 24-h Uald reflects the integrated secretion over a full day (20), effectively buffering these episodic secretory pulses. In our study population without medication adjustment, both 24-h Uald and UARR levels were higher in the PA group than in the EH group. Notably, UARR demonstrated higher specificity than ARR (0.791 vs. 0.740), further supporting the advantage of urinary markers in reducing stress interference and assessing the true biochemical load. Current guidelines recommend that all hypertensive patients should undergo at least one PA screening. Given its diagnostic stability and ease of implementation, UARR is particularly well-suited for large-scale screening in primary care settings.

After adjusting for age, BMI, renal function, serum potassium and a history of CHD, both 24-h Uald and UARR remained independently associated with PA, suggesting that they may provide supplementary information for PA screening in patients receiving antihypertensive medications. Notably, the prevalence of prior coronary artery disease and diabetes mellitus was higher in the PA group than in the EH group, and a history of CHD remained positively associated with PA in the multivariate analysis. However, the significance of this association was markedly reduced in Model 2. This finding suggests that the interpretation of CHD history should be approached with caution. It is possible that PA had been present for a prolonged period, leading to chronic aldosterone excess. Aldosterone may promote plaque formation through mechanisms such as activation of vascular endothelial cells and facilitation of inflammatory cell migration (21–23), thereby contributing to the development of CHD. However, CHD is often considered a high-risk condition, and patients with coronary artery disease are more likely to undergo PA screening, which may lead to selection bias and contribute to the higher prevalence observed in this study. Therefore, these findings suggest an association between a history of CHD and PA, but the directionality and underlying mechanisms remain difficult to ascertain.

In addition, lower eGFR levels were independently associated with PA, which is consistent with previous studies (24). Since 24-h Uald is an excretory product of renal filtration, its urinary excretion is influenced by glomerular filtration capacity (25). Moreover, chronic aldosterone excess can lead to renal structural and functional damage through proinflammatory and profibrotic mechanisms, resulting in a bidirectional interaction between aldosterone excess and renal impairment (26).Notably, the correlation analysis in this study revealed that while 24-h Uald levels exhibited a clear dependency on serum potassium and renal function, UARR demonstrated a more stable profile. Specifically, UARR showed no significant correlation with serum potassium and only a marginal correlation with eGFR. In model 3, the correlation of eGFR was still statistically significant, but the significance level was substantially reduced. This may be due to the inclusion of renin as the denominator in the UARR index, the level of which not only reflects the suppressed state of the RAAS system, but also to some extent reflects the renal perfusion and reserve function. Consequently, the incorporation of renin into the UARR index functions as a robust correction mechanism, effectively mitigating the diagnostic bias introduced by variations in renal function and electrolyte status.

A similar corrective effect was observed in the analysis of BMI. In model 2, BMI was significantly associated with PA, which is consistent with previous findings showing a positive correlation between BMI and 24-hour urinary aldosterone (27, 28). However, in model 3, after incorporating renin indicators, BMI was no longer an independent correlate of PA. Previous studies have shown that adipocytes promote aldosterone synthesis and secretion through multiple pathways, and that, in addition to traditional RAAS activation, adipokines can directly stimulate the adrenal cortex, leading to increased aldosterone levels (29, 30). Although aldosterone secretion is elevated in obese individuals, this increase is often insufficient to cause the profound renin suppression observed in PA patients; therefore, BMI is not significantly correlated with renin concentration. Consequently, by using renin as the denominator, the UARR metric effectively adjusts for obesity-mediated physiological fluctuations in aldosterone, thereby attenuating the independent predictive contribution of BMI in multivariable analyses.

Multivariable analysis showed that both 24-h Uald and UARR were independently associated with PA, but their diagnostic performance differed substantially in ROC curve analysis. In this study, the AUC of 24-h Uald alone without medication washout was only 0.657, indicating limited efficacy in distinguishing PA from EH. In contrast, the AUC of UARR reached 0.862, which was comparable to that of ARR. This result differs from the findings of You et al. (10), who reported that the AUC of 24-h Uald under the influence of medication could be as high as 0.82. The weaker performance of 24-h Uald in our study (AUC 0.657 overall and 0.688 in the false-negative drug group) may be due to differences in subgroup definitions. You et al. mainly classified drugs based on their direction of interference with renin, whereas the “false-negative group” in the present study included a complex combination of CCBs, ARBs, ACEIs, and diuretics. Although this grouping increases the heterogeneity of interfering factors, it more closely reflects the real-world scenario of multi-drug combination therapy in clinical practice and highlights the limitations of 24-h Uald for PA screening in the context of complex medication use.

UARR introduces plasma renin concentration as the denominator, which effectively mitigates the bias caused by antihypertensive drugs on the RAAS axis. In EH patients, drug-induced increases in aldosterone are usually accompanied by parallel fluctuations in renin, resulting in a relatively stable ratio. In contrast, in PA patients, even when renin-stimulating medications are administered, renin levels remain suppressed due to autonomous aldosterone secretion from the adrenal glands. In further subgroup analyses, we also found that the diagnostic AUC of UARR was higher than that of ARR, with improved specificity in patients taking medications that produce false-negative ARR results, which may further explain this observation. Therefore, UARR more sensitively reflects the underlying imbalance of the RAAS system than simple excretory metrics, and demonstrates greater diagnostic value in clinical settings with complex medication use.

The main limitation of this study is the relatively small sample size. In addition, due to the limited number of patients taking only false-positive drugs, it was difficult to evaluate the diagnostic performance in this specific subgroup. In future studies, we plan to expand the sample size and further refine subgroup classification to more comprehensively assess the clinical diagnostic efficacy of the relevant indicators.

In conclusion, this retrospective study evaluated the screening value of 24-h Uald and UARR for PA under conditions of ongoing medication use. The diagnostic performance of 24-h Uald alone was limited, whereas UARR, which incorporates renin metrics, substantially improved screening specificity. These findings provide useful evidence for PA screening in patients who are unable to discontinue antihypertensive therapy.

## Data Availability

The raw data supporting the conclusions of this article will be made available by the authors, without undue reservation.
